# IGF2BP2 Shapes the Tumor Microenvironment by Regulating Monocyte and Macrophage Recruitment in Bladder Cancer

**DOI:** 10.1002/cam4.70506

**Published:** 2024-12-23

**Authors:** Jianpeng Li, Yunzhong Jiang, Minghai Ma, Lu Wang, Minxuan Jing, Zezhong Yang, Lizhao Wang, Quanpeng Qiu, Rundong Song, Yuanchun Pu, Yuanquan Zhang, Nan Mei, Mengzhao Zhang, Jinhai Fan

**Affiliations:** ^1^ Department of Urology First Affiliated Hospital of Xi'an Jiaotong University Xi'an Shaanxi China; ^2^ Department of Breast Surgery First Affiliated Hospital of Xi'an Jiaotong University Xi'an Shaanxi China; ^3^ Department of General Surgery First Affiliated Hospital of Xi'an Jiaotong University Xi'an Shaanxi China; ^4^ Department of Hematology First Affiliated Hospital of Xi'an Jiaotong University Xi'an Shaanxi China; ^5^ Department of Vascular Surgery First Affiliated Hospital of Xi'an Jiaotong University Xi'an Shaanxi China

**Keywords:** bladder cancer, epithelial cell, IGF2BP2, m6A, macrophage, tumor infiltration immune cells

## Abstract

**Background:**

Immunotherapy has shown promise for bladder cancer (BC) treatment but is effective only in a subset of patients. Understanding the tumor microenvironment (TME) and its regulators, such as the expression of N6‐methyladenosine (m6A) regulators, may improve therapeutic outcomes. This study focuses on the role of IGF2BP2, an m6A reader, in modulating the BC TME.

**Methods:**

Transcriptomic and single‐cell RNA‐seq data from public databases were analyzed to identify BC subgroups and investigate IGF2BP2's role in the TME. Clustering and PCA identified key m6A regulators. NicheNet and SCENIC analyses were used to predict cell–cell interactions and transcriptional regulators, respectively. IGF2BP2's role in macrophage recruitment was validated via co‐culture experiments and RNA sequencing.

**Results:**

Unsupervised clustering identified BC subgroups with distinct TME characteristics, with IGF2BP2 emerging as a key regulator associated with poor prognosis and reduced response to immunotherapy. Single‐cell analysis revealed IGF2BP2's high expression in the GE‐9 epithelial subpopulation, characterized by immune evasion features and cytokine‐mediated macrophage recruitment. NicheNet analysis showed that GE‐9 cells interact with monocyte/macrophage populations through cytokine signaling. Co‐culture experiments confirmed IGF2BP2's role in recruiting macrophages, partially mediated by CCL2. Furthermore, IGF2BP2 expression was linked to immunosuppressive M2‐like and SPP1+ macrophages, contributing to an angiogenesis‐promoting and immunosuppressive TME.

**Conclusion:**

IGF2BP2 shapes the BC TME by modulating macrophage infiltration and polarization, leading to an immunosuppressive microenvironment that hinders immunotherapy. Targeting IGF2BP2 could enhance the efficacy of current therapies and improve patient outcomes.

## Graphical abstract

This study reveals that in the bladder cancer microenvironment, epithelial cells with elevated IGF2BP2 expression can recruit monocytes/macrophages. This process allows tumor cells to modify the TME and influence the response to immunotherapy.

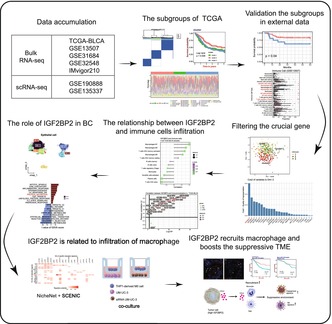

**Abbreviations**
BC: bladder cancer
BLCA: bladder cancer
GEO: Gene Expression Omnibus
GO: Gene Ontology
GSVA: gene set variation analysis
ICIs: immune checkpoint inhibitors
m6A: N6‐methyladenosine
PCA: Principal Component Analysis
scRNA‐seq: single‐cell RNA sequencing
ssGSEA: single‐sample Gene Set Enrichment Analysis
TCGA: the cancer genome atlas
TME: tumor microenvironment
tSNE: t‐distributed stochastic neighbor embedding
UMAP: uniform manifold approximation and projection

## 1 Introduction

Bladder cancer (BC) is a prevalent urogenital malignancy worldwide, with approximately 430,000 newly diagnosed cases and over 165,000 annual deaths [1]. Although there have been advancements in treatment, clinical outcomes in patients with BC remain suboptimal [2, 3]. More effective therapies need to be developed and applied.

The tumor microenvironment (TME) is a complex milieu composed of diverse cell populations, including malignant cells, immune cells, stromal cells, and other cell types [4]. During the past decade, cancer biology research has concentrated on the TME as a potential therapeutic target for drug discovery. Recent investigations underscore the influential role of the TME in dictating the progression and prognostic outlook of malignancies [5, 6]. Our previous research has demonstrated that immune cell infiltration, including in bladder and renal cell carcinoma, is closely linked to patient prognosis [7, 8].

Immunotherapy, particularly immune checkpoint blockade (ICB) targeting PD‐1/L1 and CTLA‐4, has shown promise by benefiting a subset of patients with advanced cancers. However, despite these successes, the overall survival rate remains below 30% [9, 10, 11], highlighting the limitations of current therapies and the challenges of drug resistance in certain patient populations. Therefore, investigating the TME variations is crucial for refining immunotherapeutic strategies and improving patient outcomes.

Among over 150 RNA modifications, N6‐methyladenosine (m6A) methylation is the most common in eukaryotic cells, influencing mRNA, lncRNA, and miRNA [12, 13]. Recent studies indicate that m6A modification is essential in shaping the interaction between tumor cells and their microenvironment, thereby influencing tumor progression and immune responses [14, 15, 16, 17]. Therefore, further investigation is needed to fully elucidate the association and regulatory mechanisms between immune cells and m6A modifications within the BC TME.

Insulin‐like growth factor 2 mRNA‐binding protein 2 (IGF2BP2), a pivotal player in m6A regulation, identifies and binds m6A sites on target RNAs [18]. Previous research on the IGF2BP family has predominantly centered on their roles in metabolism, mitochondrial activity, and energy storage [19, 20]. Recent experimental data underscore a connection between IGF2BP2 and the development of various cancers [19, 21], including hepatocellular carcinoma [22], breast cancer [23], ovarian cancer [24], colon cancer [25], and esophageal cancer [26]. While research has highlighted the involvement of IGF2BP2 in various cancers, its definitive role in bladder cancer and association with the immune microenvironment are not fully elucidated.

This study curated bulk RNA‐seq and single‐cell RNA sequencing (scRNA‐seq) data from public databases to explore the relationship between m6A modifications and the BC TME. Through unsupervised clustering and bioinformatics analysis, we identified IGF2BP2 as a potential key influencer on the BC TME. These findings shed light on the possible role of IGF2BP2 in shaping the TME of bladder cancer.

## 2 Methods

### 2.1 Data Accumulation

Transcriptomic and clinical data for TCGA‐BLCA were obtained from UCSC Xena (https://xenabrowser.net/datapages/), with clinical details summarized in Table S1. The IMvigor210 dataset, including transcriptomic profiles and immune checkpoint inhibitor response data, was accessed through the R package IMvigor210CoreBiologies [27]. For validation, bulk transcriptomic datasets GSE13507, GSE31684, and GSE32548 were retrieved from the Gene Expression Omnibus (GEO, https://www.ncbi.nlm.nih.gov/geo/) [28, 29, 30, 31, 32]. Single‐cell RNA‐seq data were retrieved from GSE190888 and GSE135337 datasets.

### 2.2 The Collection of m6A Regulators

In previous studies, 26 m6A regulators were identified and selected for further analysis [33, 34, 35, 36]. These regulators can be classified into three types: writers (CBLL1, VIRMA, METTL3, METTL5, METTL14, METTL16, WTAP, RBM15, RBM15B, ZNF217, ZC3H13), readers (ELAVL1, FMR1, HNRNPA2B1, HNRNPC, IGF2BP1, IGF2BP2, IGF2BP3, LRPPRC, YTHDC1, YTHDC2, YTHDF1, YTHDF2, YTHDF3), and erasers (ALKBH5, FTO). Table S1 provides more information about these genes.

### 2.3 Bioinformatics Analysis

#### 2.3.1 Clustering Analysis

Unsupervised clustering analysis, a machine learning technique, was applied to discern unique m6A modification patterns from the expression data of m6A regulator genes. “ConsensusClusterPlus” R package was used for sample classification via k‐means clustering [37]. The optimal number of clusters (k) was determined by examining the cumulative distribution function (CDF) curve and Delta area plot from “ConsensusClusterPlus.” Parameters “reps = 100” were used to ensure robust clustering. Principal Component Analysis (PCA) was utilized to identify key components, employing the “FactoMineR” R package to analyze principal factors within m6A regulator subgroups [38].

#### 2.3.2 Cluster Validation and Sample Classification in External Datasets

Cluster‐specific gene sets were constructed from the TCGA‐BLCA dataset by identifying the top 100 significantly upregulated genes for each cluster using the “limma” R package. Differential expression analysis was performed with criteria of adjusted *p* value < 0.05, ranking genes by log_2_ fold change to ensure cluster‐specificity.

To classify samples in external datasets (GSE13507, GSE31684, GSE32548, and IMvigor210), single‐sample Gene Set Enrichment Analysis (ssGSEA) was applied using these cluster‐specific gene sets. The enrichment scores for each sample were calculated across all clusters, and each sample was assigned to the cluster corresponding to its highest ssGSEA score.

#### 2.3.3 Evaluating the Immune Cell Infiltration

The infiltration of immune cells in the samples was calculated using the CIBERSORT algorithm provided in R package “cibersort” [39]. The LM22 signature matrix was used as input for CIBERSORT to deconvolute the proportions of 22 immune cell types within bulk RNA‐seq data. Immune cell fractions were compared across m6A‐based subgroups using Wilcoxon tests. The R package “estimate” was used to evaluate stromal cells and immune cells in each sample and to obtain immune and stromal scores [40].

#### 2.3.4 scRNA‐Seq Datasets Processing

Single‐cell RNA‐seq data from GSE190888 and GSE135337 were processed using the Seurat R package for quality control, integration, and downstream analysis [41]. The preprocessing workflow included the following steps. Quality Control: Low‐quality cells were filtered by excluding those with fewer than 200 detected genes or with mitochondrial gene expression exceeding 20% of total counts. Genes detected in fewer than 3 cells were removed. Batch Effect Removal and Dataset Integration: Batch effects across multiple datasets were corrected using the Harmony algorithm, ensuring a unified expression matrix for downstream analysis [42]. Dimensionality Reduction and Clustering: Principal Component Analysis (PCA) was performed on the integrated expression matrix using the RunPCA function. The top 30 principal components (PCs) were selected based on elbow plots and used for downstream clustering and visualization. Clusters were identified using the FindNeighbors and FindClusters functions with a resolution of 0.4 for epithelial cells and 0.2 for all cells. Uniform Manifold Approximation and Projection (UMAP) was applied for visualization in two dimensions using the RunUMAP function with the same PCs and default parameters.

#### 2.3.5 CNV Estimation

The inferCNV package (https://github.com/broadinstitute/infercnv) was used to detect copy number variations (CNVs) in epithelial cells and identify malignant cells. To accommodate the absence of a clearly defined reference group, a reference‐free mode was applied during the analysis. The CNV score for each cell was defined as the sum of the inferred CNV values across all genes within that cell. This approach allowed us to identify malignant cells based on their deviating CNV profiles relative to the baseline.

#### 2.3.6 Enrichment Analysis

To investigate pathway‐level differences between subgroups of epithelial cells, we performed gene set variation analysis (GSVA) using the “GSVA” R package [43]. Normalized gene expression data from the Seurat object were used as input, and the hallmark gene sets (h.all.v7.1.symbols.gmt) served as the reference. Enrichment scores were computed with the GSVA function, using “Gaussian” kernel estimation to account for the continuous expression data.

Differentially enriched pathways between subgroups of cells were identified using a linear model implemented in the limma package. A design matrix was constructed, and contrasts were defined to compare GSVA scores between the two groups. Pathways with an adjusted *P* value < 0.05 (Benjamini‐Hochberg correction) were considered significant. The top 10 enriched pathways for each group, ranked by absolute *t*‐value, were visualized as bar plots.

#### 2.3.7 Nichenet Analysis

Cell–cell communication within the bladder cancer TME was analyzed using the NicheNet R package (version 2.1.5). Single‐cell RNA‐seq data from GSE190888 and GSE135337 were used to define the epithelial cell subgroup as the receiver, while immune cells were identified as senders. Differentially expressed genes (DEGs) in GE‐9 and genes expressed in at least 10% of the cells were selected as the background gene set. NicheNet's ligand‐receptor network and ligand‐target matrix were used to predict active ligand‐receptor pairs and rank ligands based on their regulatory potential for the GE‐9‐specific gene set. Significant interactions were visualized through heatmaps.

#### 2.3.8 SCENIC Analysis

Transcription factor (TF) activity was analyzed using pySCENIC, a Python implementation of the SCENIC pipeline, based on raw count matrices from single‐cell RNA‐seq datasets GSE190888 and GSE135337. Regulatory relationships between TFs and target genes were inferred using GRNBoost2, followed by motif enrichment analysis with RcisTarget to identify enriched TF binding motifs and define regulons comprising TFs and their direct target genes. The activity of each regulon in individual cells was quantified using AUCell, which generated a regulon activity matrix. To identify key TFs in the GE‐9 epithelial cell subgroup, differentially activated regulons were determined using the Wilcoxon rank‐sum test, with multiple testing correction performed via the Benjamini‐Hochberg method.

### 2.4 Cell and Molecular Biology Analysis

#### 2.4.1 Cell Culture and Cell Transfection

In this study, two cell lines (UM‐UC‐3, THP‐1) were utilized, all obtained from the Wuhan Pricella Biotechnology. IGF2BP2‐knockdown in UM‐UC‐3 was achieved using siRNA (sequences in Table S3) and GP‐transfect‐mate (GenePharma, Shanghai).

#### 2.4.2 RNA Extraction and RT‐qPCR

Total RNA extraction from cultured cells was performed using the RNAfast200 kit (Feijie, Shanghai), following the manufacturer's instructions. Relative gene expression levels were calculated using the 2^−ΔΔCt^ method, normalizing to the internal reference gene GAPDH.

#### 2.4.3 Protein Extraction and Western Blotting

Total proteins were extracted using RIPA buffer with protease and phosphatase inhibitors. After 10% SDS‐PAGE separation, proteins were transferred to PVDF membranes, blocked with 5% skim milk, and incubated with primary antibodies (IGF2BP2 and β‐actin) overnight at 4°C. Post‐TBST washes, membranes were incubated with secondary antibodies, and protein bands were visualized using Bio‐Rad's ECL system. The following antibodies were used: IGF2BP2 (11601‐1‐AP; Proteintech) and β‐actin (AC026; ABclonal) antibodies.

#### 2.4.4 Transwell Assay

In the Transwell assay, UM‐UC‐3, and siRNA‐treated cells were seeded in the upper chamber, above a lower chamber containing 10% FBS medium. After 24 h, cells in the lower chamber were PBS‐washed, fixed with 4% paraformaldehyde, and stained with crystal violet. Images were captured using an inverted microscope at 200× magnification.

#### 2.4.5 Immunofluorescence

Bladder cancer tissues from the First Affiliated Hospital of Xi'an Jiaotong University were used for immunofluorescence. The process involved deparaffinization, antigen retrieval, and blocking with goat serum. Sections were incubated with primary antibodies, followed by CY3‐conjugated secondary antibodies (1:300; Servicebio, GB21303) and DAPI counterstaining. After washing and autofluorescence quenching, slides were mounted with anti‐fading medium.

The following antibodies were used to detect specific proteins: anti‐CD14 (rabbit, 1:500, servicebio, GB113374), anti‐CD68 (rabbit, 1:500, servicebio, GB113150), anti‐IGF2BP2 (rabbit, 1:200, Proteintech, 11,601‐1‐AP).

### 2.5 Statistical Analysis

R software (version 4.3.0; The R Foundation for Statistical Computing) and graphpad prism 8.0 were used for data analysis and statistics. The Wilcoxon test was used to compare the expression of m6A genes in different groups. Kaplan–Meier survival analysis and log‐rank tests were utilized for analyzing OS in different subgroups [44]. Spearman correlation evaluated the correlation between m6A genes and tumor‐infiltrating immune cells. All statistical tests were two‐sided, with significance at *p* < 0.05.

## 3 Results

### 3.1 The Landscape of m6A Genes in BC Revealed by Bulk RNA‐Seq

**FIGURE 1:**
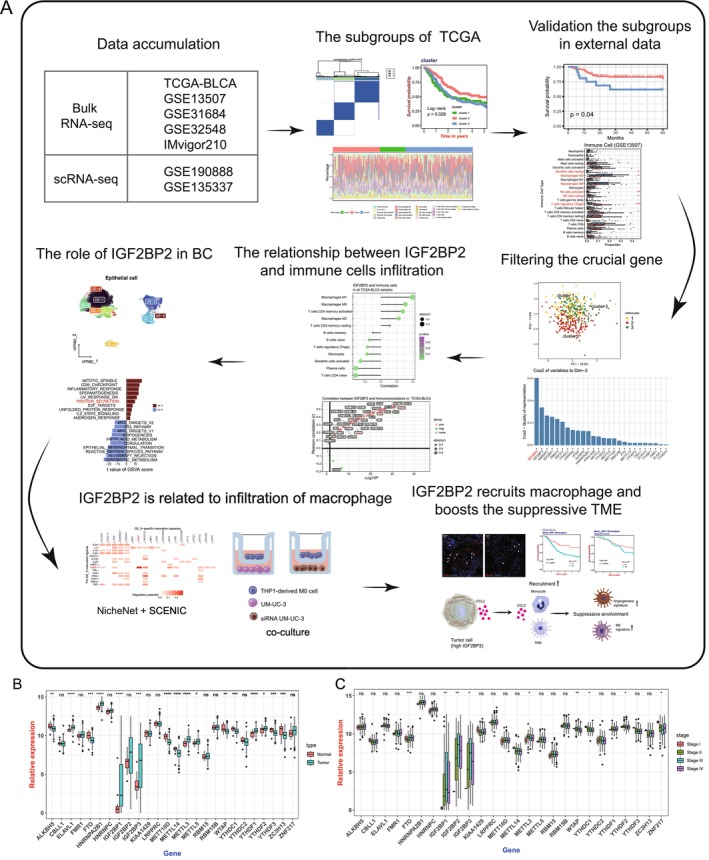
Study workflow and expression landscape of m6A regulators in bladder cancer. (A) Flowchart illustrating the sequential steps of this study. (B) Boxplot comparing the expression levels of m6A regulatory genes between tumor tissues and adjacent normal tissues. (C) Boxplot showing the differential expression of m6A regulatory genes across bladder cancer stages. Statistical significance is indicated: *p < 0.05, **p < 0.01, ***p < 0.001.

The flowchart depicted in Figure 1A illustrates the sequential process of this study. Among the 407 patients diagnosed with BC, the expression of “readers,” “writers,” and “erasers” are correlated with various clinical characteristics. Specifically, ALKBH5, ELAVL1, FTO, HNRNPA2B1, IGF2BP1, IGF2BP3, METT10D, METTL14, METTL3, METTL5, WTAP, YTHDC1, YTHDF1, YTHDF2, YTHDF3, and ZC3H13 displayed differential expression in tumor and normal tissues (Figure 1B). FTO, IGF2BP1, IGF2BP2, IGF2BP3, METTL3, WTAP, YTHDC1, YTHDF2, and ZNF217 exhibited distinct expression patterns across different pathological stages (Figure 1C). These findings suggest that m6A regulators significantly influence the BC microenvironment and warrant further analysis.

### 3.2 Validation and Prognostic Significance of BC Subgroups Based on m6A Regulators

**FIGURE 2:**
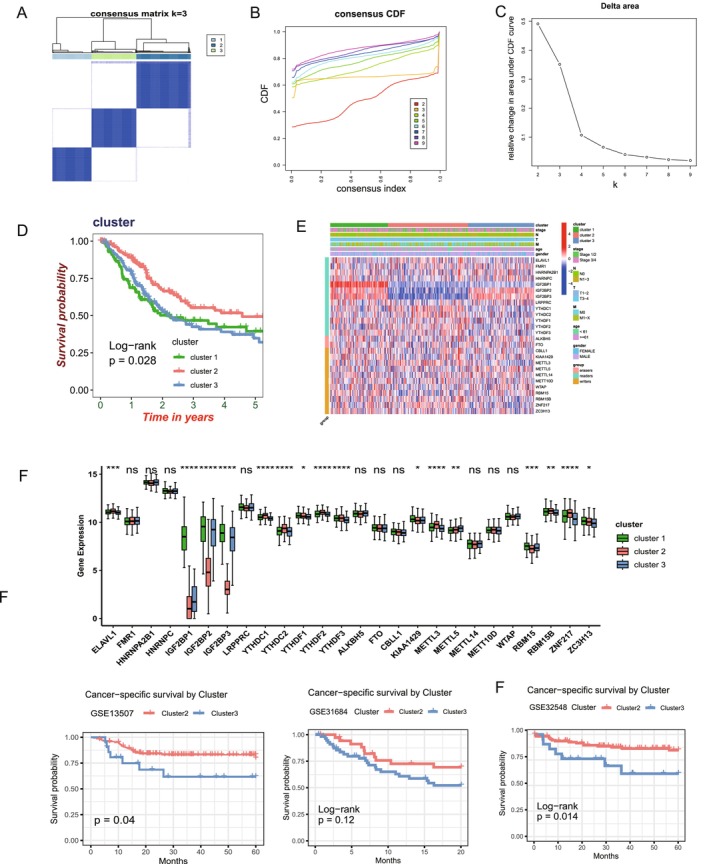
Stratification of bladder cancer subgroups and their prognostic significance based on m6A regulator expression. (A) Consensus clustering matrix for *k* = 3, showing robust subgroup classification based on m6A regulator expression profiles in the TCGA‐BLCA cohort. (B) Cumulative distribution function (CDF) curve and (C) delta area plot illustrating the optimal number of clusters (*k* = 3) with minimal variation in consensus index. (D) Kaplan–Meier survival curves for the three m6A‐based subgroups, showing significant differences in overall survival (Log‐rank *p* < 0.05). (E) Heatmap of m6A regulator expression profiles across the three clusters, highlighting distinct expression patterns. (F) Boxplot showing the differential expression of IGF2BP2 and IGF2BP3, with elevated levels observed in Clusters 1 and 3, associated with poorer prognosis. Statistical significance is indicated: *p < 0.05, **p < 0.01, ***p < 0.001.

To investigate whether m6A regulators could stratify BC patients into clinically relevant subgroups, we performed unsupervised clustering using the “ConsensusClusterPlus” R package. This analysis, applied to the expression profiles of m6A regulators in the TCGA‐BLCA cohort, identified three robust clusters (*k* = 3; Figure 2A–C). Kaplan–Meier survival analysis demonstrated that patients in Cluster 2 had significantly better prognosis compared to those in Clusters 1 and 3 (Figure 2D). Differential expression analysis of m6A regulators among the three clusters revealed significant variation in the expression of 16 genes (Figure 2E). Among these, IGF2BP2 and IGF2BP3 were notably overexpressed in Clusters 1 and 3 (Figure 2F), which corresponded to the poorer prognosis observed in these subgroups. These findings suggest that m6A regulators play a critical role in BC progression and may serve as biomarkers for clinically relevant subtyping.

To further validate this classification scheme, we applied the cluster‐specific marker genes derived from the TCGA‐BLCA cohort (Table S4) to several independent GEO datasets. Enrichment scores for these marker genes were calculated using Gene Set Variation Analysis (GSVA), enabling the assignment of cluster labels to each sample. Consistent with the TCGA findings, survival analysis in GSE13507, GSE31684, and GSE32548 demonstrated that patients in Cluster 2 consistently exhibited significantly better prognosis compared to those in Clusters 1 and 3. These results confirm the robustness and clinical relevance of the m6A‐based subtypes, supporting their utility in BC patient stratification and prognosis.

### 3.3 Distinct Immune Infiltration Characteristics of m6A‐Based BC Subgroups

**FIGURE 3:**
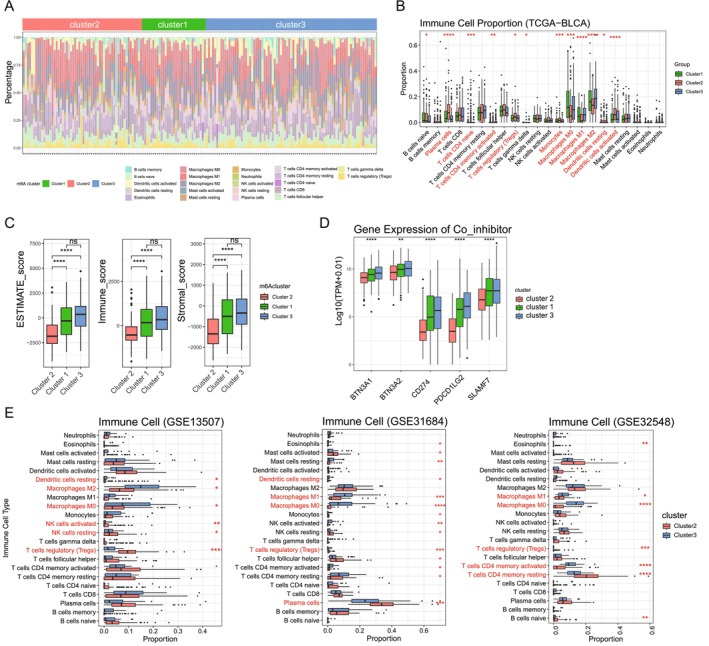
Distinct immune infiltration characteristics across m6A‐based bladder cancer subgroups. (A) Proportion of 22 immune cell types among the three m6A‐based subgroups in the TCGA‐BLCA cohort, estimated using the CIBERSORT algorithm. (B) Boxplot showing the proportions of 22 immune cell types across the three m6A‐based subgroups (Cluster 1, Cluster 2, and Cluster 3) in the TCGA‐BLCA cohort, as estimated using the CIBERSORT algorithm. (C) ESTIMATEScore, ImmuneScore, and StromalScore comparisons among the three subgroups, demonstrating significant differences in tumor microenvironment composition. (D) Differential expression of co‐inhibitory molecules and cell adhesion genes among the three subgroups. (E) Immune cell infiltration patterns in the external GSE13507, GSE31684, and GSE32548 datasets, showing consistent trends with TCGA findings. Statistical significance is indicated: *p < 0.05, **p < 0.01, ***p < 0.001.

Building on the identified m6A‐based subgroups, we next explored their relationship with the TME. Using the LM22 immune signature matrix and the CIBERSORT algorithm, we estimated the proportions of 22 immune cell types in TCGA‐BLCA samples. The results revealed distinct immune infiltration patterns among the subgroups, particularly between Cluster 1/3 and Cluster 2 (Figure 3A). Specifically, the infiltration levels of T cells, macrophages, and monocytes were significantly different across the subgroups, highlighting the immune heterogeneity of these clusters (Figure 3B). To further characterize the immune and stromal composition, we calculated ESTIMATEScore, ImmuneScore, and StromalScore for each subgroup using the “estimate” R package. Consistent with the CIBERSORT results, Cluster 2 exhibited significantly lower ImmuneScore and StromalScore compared to Clusters 1 and 3 (Figure 3C), suggesting that Cluster 2 is associated with a higher tumor purity and a distinct TME composition. These findings underscore the unique immune microenvironment features of each m6A‐based BC subgroup.

In addition to immune infiltration patterns, we analyzed the expression of co‐inhibitory and cell adhesion molecules among the subgroups, which revealed significant differences in their expression profiles (Figure 3D). Cluster 1 and Cluster 3 exhibited elevated expression of co‐inhibitory molecules, including PD‐L1 (CD274) and PDCD1LG2 (PD‐L2), compared to Cluster 2. These molecules are critical for suppressing T‐cell activation and promoting immune evasion, suggesting a more immunosuppressive environment in Clusters 1 and 3. In contrast, Cluster 2 displayed reduced expression of these co‐inhibitory molecules, potentially reflecting a less immunosuppressive or more immune‐active environment. Similarly, cell adhesion molecules, such as ICAM1 and SELP, also showed distinct expression patterns across the subgroups, implying potential differences in immune cell recruitment and interaction within the tumor microenvironment.

To further validate the immune infiltration characteristics observed in TCGA‐BLCA, we extended our analysis to three independent external cohorts (GSE13507, GSE31684, and GSE32548) using the LM22 immune signature matrix and the CIBERSORT algorithm. Consistent with the TCGA‐BLCA findings, significant differences in immune cell infiltration patterns were observed between the m6A‐based subgroups, particularly between Cluster 2 and Cluster 3 (Figure 3E). Across all three datasets, Cluster 2 exhibited elevated infiltration of M2 macrophages, Tregs, and activated dendritic cells, suggesting a more immunosuppressive microenvironment. These findings align with the immune heterogeneity initially identified in TCGA‐BLCA and reinforce the immunological divergence between these m6A‐based subgroups. This validation in independent cohorts underscores the robustness and biological relevance of the subgroup classifications. Together, these findings suggest that immune modulation mechanisms vary significantly across m6A‐based subgroups, contributing to their distinct prognostic outcomes and immune profiles.

### 3.4 IGF2BP2 Emerges as a Crucial Gene in BC Subgroups and TME

**FIGURE 4:**
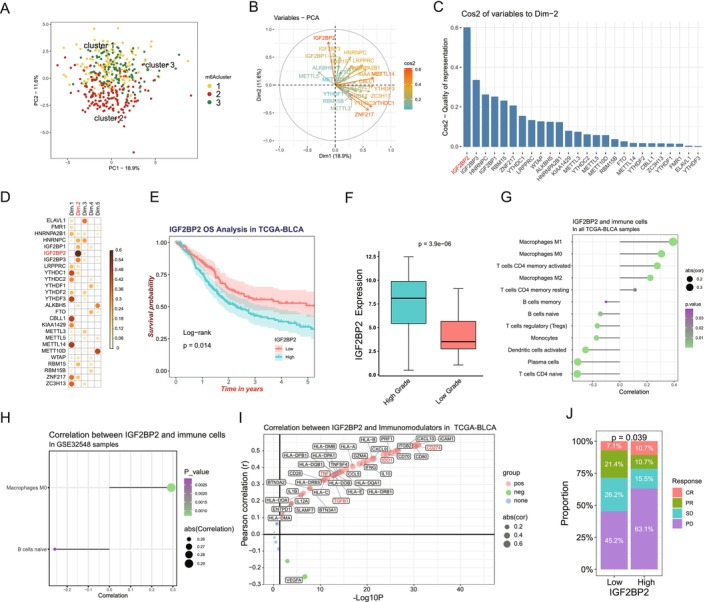
IGF2BP2 as a crucial factor influencing m6A‐based subgroups and immune microenvironment in bladder cancer. (A) Principal Component Analysis (PCA) of m6A regulators in TCGA‐BLCA samples, showing distinct clustering of the three m6A‐based subgroups (Cluster 1, Cluster 2, and Cluster 3) along PC1 and PC2. (B) PCA loading plot identifying IGF2BP2 as a top contributor to PC2 variance. (C) Quality of representation (Cos2) of m6A regulators to PCA dimensions, highlighting the prominent role of IGF2BP2. (D) Contribution of m6A regulators across the first five PCA dimensions, with IGF2BP2 showing significant influence. (E) Kaplan–Meier survival analysis of IGF2BP2 expression levels in TCGA‐BLCA, demonstrating poorer survival in patients with high IGF2BP2 expression. (F) Boxplot of IGF2BP2 expression in high‐grade versus low‐grade tumors in TCGA‐BLCA, showing significantly higher expression in high‐grade tumors. (G) Correlation between IGF2BP2 expression and immune cells in TCGA‐BLCA. (H) Validation of IGF2BP2's association with immune cell infiltration in the GSE32548 dataset. (I) The correlation between IGF2BP2 expression and immunomodulatory molecules in TCGA‐BLCA. (J) Proportions of immunotherapy response categories (CR, PR, SD, and PD) in the IMvigor210 cohort stratified by IGF2BP2 expression, indicating poorer response in the high‐expression group.

To identify m6A regulators with the most significant influence on subgroup differentiation, we conducted PCA on the expression profiles of m6A regulators across BC subgroups. As shown in Figure 4A, PCA projected BC subgroups (Cluster 1, 2, and 3) with clear separation along PC2, which captures significant variance related to subgroup differentiation. IGF2BP2 emerged as the top contributor to PC2, supported by its prominent loading vector (Figure 4B) and highest Cos2 value (Figure 4C). Further analysis across five dimensions (Figure 4D) confirmed its dominant role in subgroup differentiation and its broader influence across multiple principal components. These findings underscore IGF2BP2 as a pivotal factor in BC subgrouping and TME regulation.

Building on this identification, we explored the clinical and immunological significance of IGF2BP2 across multiple datasets. In the TCGA‐BLCA cohort, patients with high IGF2BP2 expression exhibited significantly poorer overall survival (*p* = 0.014, Figure 4E) and higher expression in high‐grade tumors (*p* = 3.9e‐06, Figure 4F), linking it to tumor aggressiveness. Correlation analysis revealed significant associations between IGF2BP2 and immunosuppressive cells, including M0/M2 macrophages, Tregs, and activated CD4+ memory T cells (Figure 4G). This association was validated in the GSE32548 (Figure 4H), further highlighting its role in macrophage infiltration.

**FIGURE 5:**
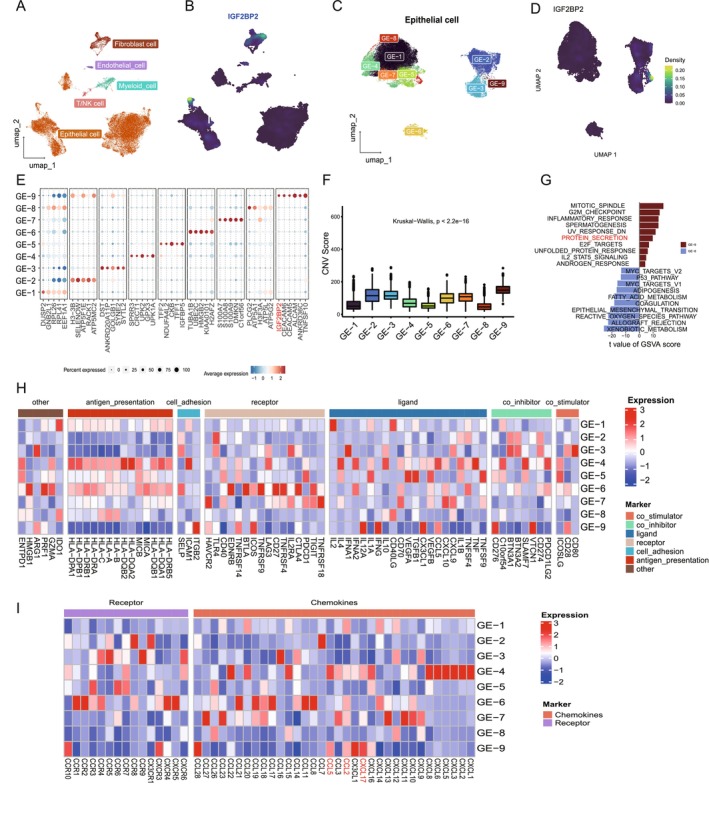
Distinct expression patterns of IGF2BP2 in epithelial cell subpopulations and its association with immune evasion mechanisms. (A) UMAP plot showing the classification of single cells into major cell types, including epithelial, myeloid, T/NK, endothelial, and fibroblast cells. IGF2BP2 expression is predominantly enriched in epithelial cells. (B) UMAP plot displaying the density of IGF2BP2 expression across epithelial cells. High IGF2BP2 expression is concentrated in specific regions, corresponding to the epithelial cells and fibroblast cells. (C) UMAP plot of epithelial cells revealing 9 subpopulations (GE‐1 to GE‐9) based on gene expression profiles. (D) IGF2BP2 is highly expressed in the GE‐9 subpopulation. (E) Dot plot showing the density of IGF2BP2 expression across epithelial subpopulations, with the highest expression in GE‐9. (F) Bar plot of CNV scores among epithelial subpopulations, indicating significantly higher CNV levels in GE‐9 compared to other subpopulations. (G) Barplot of hallmark pathway enrichment in GE‐9, showing upregulation of pathways associated with immune evasion and tumor progression, including inflammatory response, protein secretion, and G2M checkpoint. (H) Heatmap depicting the differential expression of immunomodulatory molecules (e.g., co‐stimulators, co‐inhibitors, ligands, receptors) across epithelial subpopulations. GE‐9 exhibits downregulation of antigen‐presentation molecules and upregulation of chemokines. (I) Heatmap of chemokine and receptor expression across epithelial subpopulations, highlighting upregulation of CCL2 and CXCL17 in GE‐9, which are involved in macrophage recruitment and immune suppression.

IGF2BP2 was also positively correlated with key immune checkpoint molecules (PD‐L1, IDO1) and immunosuppressive cytokines (TGFB1) in TCGA‐BLCA (Figure 4I), suggesting its involvement in immune evasion. Finally, in the IMvigor210 cohort, patients with high IGF2BP2 expression exhibited poorer responses to immune checkpoint inhibitors (*p* = 0.039, Figure 5J), underscoring its potential as a biomarker for predicting immunotherapy efficacy. Collectively, these results establish IGF2BP2 as a critical m6A regulator influencing BC subgroups, the tumor microenvironment, and clinical outcomes, highlighting its potential as a biomarker for therapeutic stratification in bladder cancer.

### 3.5 IGF2BP2 Displays Distinct Expression Pattern in Epithelial Cells

Motivated by findings from the IMvigor210 cohort, which revealed a correlation between IGF2BP2 and the immune response, we focused on examining the expression patterns of IGF2BP2 across specific cell types. To achieve this, we incorporated 11 single‐cell RNA‐sequencing samples from the GSE190888 and GSE135337 datasets (Figure S1A). Following batch correction, we visualized the distribution of cells across samples and categorized them into epithelial, endothelial, immune, fibroblast, and muscle cells based on classical marker genes (Figure 5A; Figure S1B,C; Table S5). This analysis revealed that IGF2BP2 expression is predominantly enriched in epithelial and fibroblast cells (Figure 5B), prompting us to focus further on epithelial cells for subsequent analysis.

After isolating epithelial cells and performing dimensionality reduction and clustering, we identified 10 distinct epithelial subpopulations, which were named gene element (GE), from GE‐0 to GE‐9, based on their gene expression profiles (Figure 5C). Each subpopulation exhibited unique marker genes, with significantly distinct transcriptional characteristics (Figure 5E). Notably, IGF2BP2 was highly expressed in the GE‐9 subpopulation (Figure 5D), establishing it as a marker gene for GE‐9 cells. To further characterize epithelial subpopulations, we calculated CNV scores using inferCNV. GE‐9 cells displayed significantly higher CNV scores compared to other epithelial subpopulations, particularly in contrast to the GE‐8 subpopulation, which exhibited low CNV scores (Figure 5F; Figure S1D). This result suggests that GE‐9 represents a malignant epithelial subpopulation associated with bladder cancer progression. We further compared the differentially expressed genes (DEGs) between GE‐9 and GE‐8 cells. GSVA using “hallmark gene sets” revealed that GE‐9 cells were significantly enriched in pathways related to protein secretion, inflammatory response, and G2M checkpoint (Figure 5G). These findings highlight GE‐9 as a highly proliferative and biologically active cell population that plays a critical role in bladder cancer progression.

Considering the established connection between IGF2BP2 and immune responses, we investigated immune‐related molecular features within epithelial subpopulations. Interestingly, GE‐9 cells exhibited significantly lower expression of MHC class I and class II molecules, including HLA‐A, HLA‐B, HLA‐C, HLA‐DRA, HLA‐DRB1, and others, compared to other subpopulations (Figure 5H). These molecules are key players in antigen presentation and immune surveillance, suggesting that GE‐9 cells may possess enhanced immune evasion capabilities. Furthermore, analysis of chemokine expression revealed that CCL2 and CXCL17 were significantly upregulated in GE‐9 compared to other subpopulations (Figure 5I): CCL2 is a critical chemokine for monocyte and macrophage recruitment. CXCL17 has been associated with immune suppression and recruitment of tumor‐associated macrophages (TAMs). These findings suggest that while GE‐9 cells may evade adaptive immune responses due to MHC molecule downregulation, they likely shape the tumor immune microenvironment by secreting chemokines to recruit immune cells, particularly macrophages. This dual mechanism highlights GE‐9 as a unique epithelial subpopulation actively involved in both immune modulation and tumor progression.

### 3.6 IGF2BP2 Is Related to Infiltration of Macrophages and Monocyte

**FIGURE 6:**
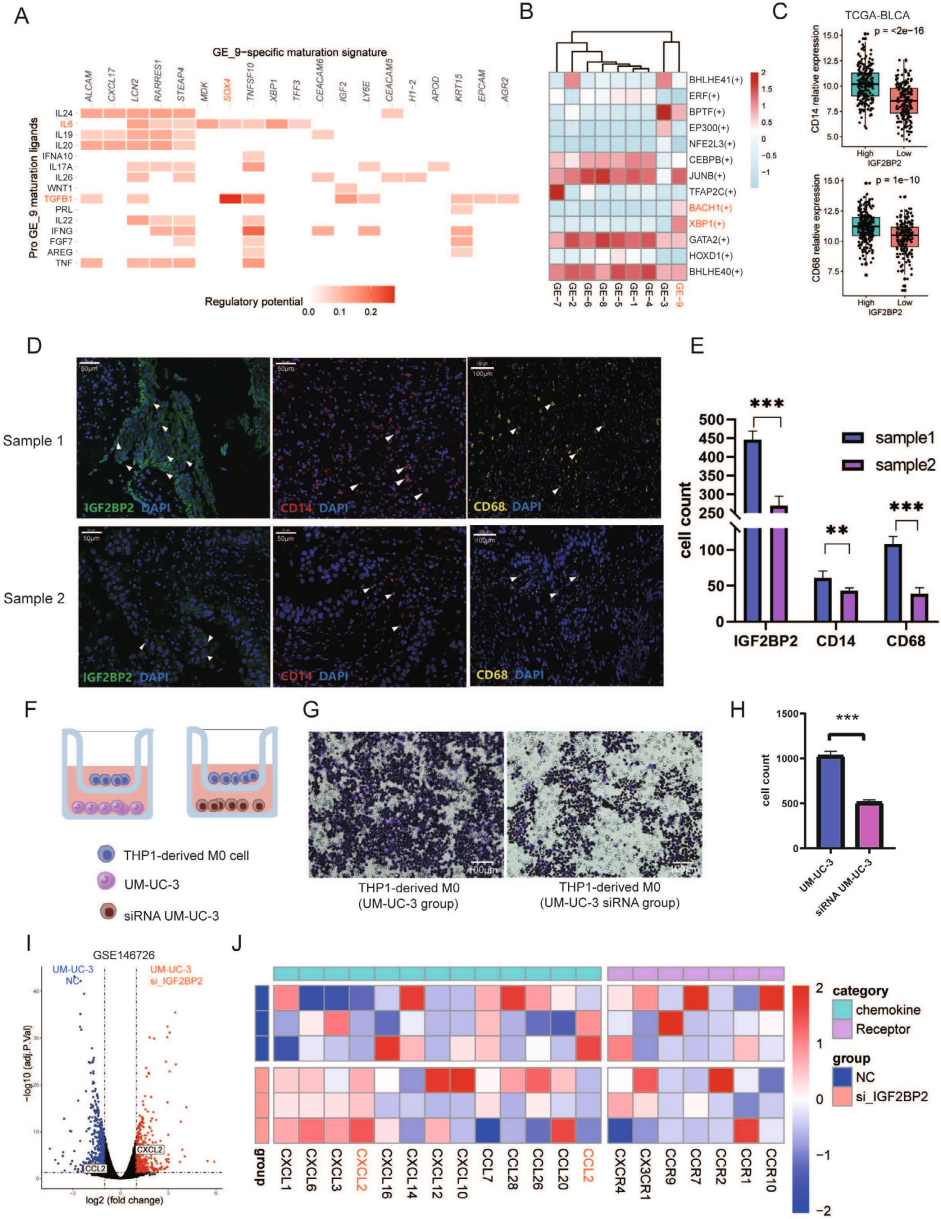
IGF2BP2's role in macrophage and monocyte infiltration within the bladder cancer tumor microenvironment. (A) NicheNet analysis showing significant ligand‐receptor interactions between epithelial cells with high IGF2BP2 expression and monocyte/macrophage populations. (B) SCENIC analysis of transcription factor activity in epithelial cells. (C) Correlation analysis of IGF2BP2 expression and monocyte/macrophage markers (CD14 and CD68) in TCGA‐BLCA, demonstrating a positive relationship. (D, E) Immunofluorescence staining of bladder cancer tissues confirming the association between high IGF2BP2 expression and increased presence of CD14+ and CD68+ cells. (F) Schematic representation of the co‐culture assay setup. THP‐1‐derived M0 macrophages were placed in the upper chamber, and UM‐UC‐3 cells (wild‐type or IGF2BP2 siRNA‐treated) were cultured in the lower chamber to assess macrophage migration. (G) Representative images of migrated M0 macrophages in the lower chamber, showing a higher number of cells in the UM‐UC‐3 group compared to the siRNA‐treated UM‐UC‐3 group. Scale bar: 100 μm. (H) Quantification of migrated M0 macrophages, demonstrating significantly reduced migration in the IGF2BP2 siRNA‐treated group compared to the wild‐type UM‐UC‐3 group. Statistical significance: ***p < 0.001. (I) Volcano plot of RNA sequencing results comparing gene expression between IGF2BP2 siRNA‐treated and wild‐type UM‐UC‐3 cells. (J) Heatmap showing differential expression of chemokines and their receptors in IGF2BP2 siRNA‐treated versus wild‐type UM‐UC‐3 cells.

Given the potential role of GE‐9 in influencing the surrounding TME, we hypothesized that GE‐9 might engage in crosstalk with other cell types in the TME. To explore the potential crosstalk between epithelial cells and immune cells in the BC TME, we utilized the “Nichenet” R package. This analysis revealed prominent ligand‐receptor interactions, including SOX4‐IL6 and SOX4‐TGFB1 pathways (Figure 6A). These pathways are well‐documented to influence macrophage recruitment and polarization, with TGFB1 particularly implicated in driving macrophages toward the M2 immunosuppressive phenotype [45]. Additionally, transcription factor activity in epithelial cells was assessed using SCENIC. Among the top transcription factors identified, BACH1 and XBP1 showed significantly higher activity in GE‐9 cells (Figure 6B). Notably, BACH1 is associated with the regulation of chemokine expression, including CCL2 [46], and XBP1 has been implicated in macrophage recruitment and immune suppression in the TME [47]. These findings strongly suggest that GE‐9 cells could influence monocyte/macrophage recruitment and activity through chemokine secretion.

To further investigate the relationship between IGF2BP2 and macrophages, we analyzed the TCGA‐BLCA dataset. A significant positive correlation was observed between IGF2BP2 expression and monocyte/macrophage markers, including CD14 and CD68 (Figure 6C). Moreover, immunofluorescence performed on bladder cancer tissue samples revealed that samples with high IGF2BP2 expression exhibited relatively high levels of CD14 and CD68, consistent with the TCGA‐BLCA and spatial transcriptomics findings (Figure 6D,E). These results collectively indicate that IGF2BP2 expression is associated with monocyte and macrophage infiltration in the bladder cancer TME.

To validate the role of IGF2BP2 in macrophage recruitment, we conducted a co‐culture assay using THP‐1‐derived M0 macrophages and UM‐UC‐3 bladder cancer cells (Figure 6F). In this setup, M0 macrophages were placed in the upper chamber while UM‐UC‐3 cells, either wild‐type or IGF2BP2 siRNA‐treated, were cultured in the lower chamber. Migrated macrophages in the lower chamber were visualized and quantified. The results demonstrated a significantly higher number of migrating macrophages in the wild‐type UM‐UC‐3 group compared to the IGF2BP2 siRNA‐treated group (Figure 6G,H). These findings provide strong evidence that high IGF2BP2 expression enhances macrophage recruitment. To elucidate the underlying molecular mechanism, we performed RNA sequencing analysis (GSE146726) on IGF2BP2 siRNA‐treated and wild‐type UM‐UC‐3 cells. The analysis revealed that the expression of CCL2, a chemokine known to play a crucial role in monocyte/macrophage recruitment, was significantly reduced in IGF2BP2 knockdown cells (Figure 6I,J). Additionally, a heatmap analysis highlighted broader changes in chemokine and receptor expression profiles, with CCL2 being prominently downregulated in the absence of IGF2BP2. These results suggest that IGF2BP2 promotes macrophage recruitment by regulating the secretion of CCL2, thus playing a critical role in shaping the immune microenvironment of BC.

### 3.7 IGF2BP2 Recruit Macrophages and Boosts the Suppressive Tumor Microenvironment

**FIGURE 7:**
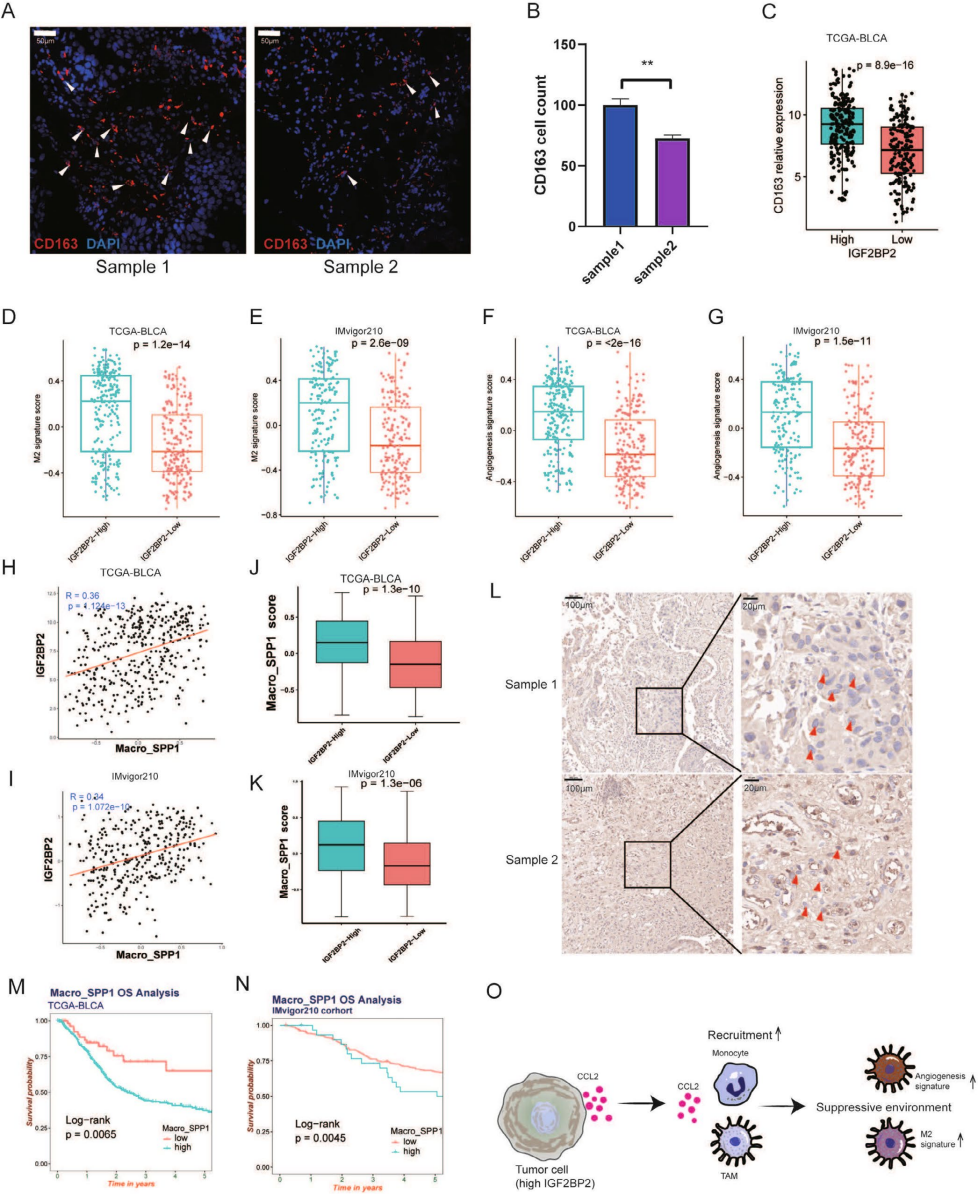
IGF2BP2‐mediated macrophage recruitment drives an immunosuppressive tumor microenvironment in bladder cancer. (A) Representative immunofluorescence images of bladder cancer tissue samples stained for CD163 (red), a marker of M2 macrophages, and DAPI (blue) for nuclei. Samples with high IGF2BP2 expression exhibit increased CD163+ cell density. Scale bar: 50 μm. (B) Quantification of CD163+ cells in two bladder cancer samples with different IGF2BP2 expression levels, showing significantly higher counts in the high‐IGF2BP2 sample. Statistical significance: **p < 0.01. (C) Boxplot of CD163 relative expression levels in high‐ and low‐IGF2BP2 expression groups in the TCGA‐BLCA dataset. High IGF2BP2 expression is associated with significantly elevated CD163 levels (*p* = 8.9e‐16). (D, E) M2 macrophage signature scores calculated using GSVA for the TCGA‐BLCA (D) and IMvigor210 (E) datasets. (F, G) Angiogenesis signature scores calculated using GSVA for the TCGA‐BLCA (F) and IMvigor210 (G) datasets. (H, I) Correlation analysis between IGF2BP2 expression and macro_SPP1 scores (SPP1+ macrophages) in TCGA‐BLCA (H) and IMvigor210 (I) datasets. (J, K) Boxplots comparing macro_SPP1 scores in high‐ and low‐IGF2BP2 expression groups in TCGA‐BLCA (J) and IMvigor210 (K) datasets. (L) Representative IHC images of bladder cancer tissue samples showing SPP1 expression. Samples with high IGF2BP2 expression demonstrate increased SPP1+ cell density compared to low IGF2BP2 samples. Scale bars: 100 μm (left) and 20 μm (right). (M, N) Kaplan–Meier survival curves for overall survival (OS) based on macro_SPP1 scores in TCGA‐BLCA (M) and IMvigor210 (N) datasets. (O) Schematic model illustrating the recruitment of SPP1+ macrophages by tumor cells with high IGF2BP2 expression via CCL2 secretion. These macrophages contribute to an angiogenic and immunosuppressive tumor microenvironment, characterized by elevated M2 and angiogenesis signatures, potentially affecting patient outcomes.

To better understand the macrophages recruited by epithelial cells with high IGF2BP2 expression, we performed immunofluorescence staining for CD163, a marker of M2 macrophages, and found that CD163 expression was positively correlated with IGF2BP2 (Figure 7A,B). Moreover, we found that the expression of CD163 is significantly high in high‐IGF2BP2 group in TCGA‐BLCA (Figure 7C), consistent with the immunofluorescence result. Given that M2‐polarized macrophages are known to facilitate angiogenesis and tumor progression [48], we sought to further investigate the potential role of M2‐like tumor‐associated macrophages (TAMs) in our dataset. To explore this, we conducted a GSVA analysis, revealing that in both the TCGA‐BLCA and IMvigor210 datasets, the high IGF2BP2 expression group had a significantly higher M2 signature score (Figure 7D,E). In addition, this group also exhibited an elevated angiogenesis score (Figure 7F,G), aligning with the known role of M2 macrophages in promoting angiogenesis. These findings suggest that tumor cells with high IGF2BP2 expression may recruit and activate M2‐like TAMs, which in turn promote angiogenesis, thus contributing to tumor progression.

However, recent studies have revealed that macrophage populations in the TME are far more complex than the traditional M1/M2 polarization model suggests [49]. For example, angiogenesis‐associated macrophages, which have been linked to poor prognosis, represent a distinct subpopulation beyond the traditional M1/M2 paradigm. Both Cheng et al. and Zhang et al. have reported that SPP1+ TAMs, a subset of angiogenesis‐associated macrophages, exhibit pro‐angiogenic properties and are correlated with worse clinical outcomes [50, 51]. In light of these findings, we extended our analysis to include SPP1+ macrophages to further delineate their relationship with IGF2BP2 expression in BC. Therefore, we further analyzed the macro_SPP1 scores in TCGA‐BLCA and IMvigor210 datasets and discovered a significant correlation between macro_SPP1 and IGF2BP2 expression (Figure 7H,I). The macro_SPP1 scores were notably higher in the high IGF2BP2 group compared to the low IGF2BP2 group (Figure 7J,K). IHC also showed higher SPP1 expression in the high IGF2BP2 sample (Figure 7L). Furthermore, survival analysis indicated that the group with high macro_SPP1 scores had poorer OS in TCGA‐BLCA and IMvigor210 cohort (Figure 7M,N). In summary, these findings indicate that tumor cells with high IGF2BP2 expression recruit both M2‐like and SPP1+ macrophages (Figure 7O), which together may contribute to an immunosuppressive microenvironment and affect treatment outcomes.

## 4 Discussion

Immunotherapy offers significant clinical benefits for patients with advanced or treatment‐resistant BC, providing some with prolonged survival [52, 53, 54]. However, not all BC patients respond effectively, and understanding the TME is essential for uncovering the molecular mechanisms underlying immunotherapy resistance. Emerging evidence suggests that specific molecules within the TME, such as IGF2BP2, may play a critical role in modulating immune cell infiltration and influencing treatment outcomes. In this study, using RNA‐seq and scRNA‐seq, we demonstrated that IGF2BP2 is linked to immune cell infiltration and identified specific macrophage subtypes recruited by IGF2BP2‐expressing epithelial cells.

Our analysis identified IGF2BP2 as a key factor differentiating the TCGA‐BLCA subgroups, with PCA highlighting its dominant contribution to subgroup‐specific variance. Given the better overall survival (OS) observed in cluster 2 and its distinct immune cell infiltration and immune checkpoint gene expression patterns, IGF2BP2 emerged as a potential regulator influencing these phenotypes. In survival analysis, higher IGF2BP2 expression was associated with poorer OS and was enriched in high‐grade tumors, highlighting its prognostic significance in BC. Moreover, correlation analysis in TCGA‐BLCA demonstrated a strong association between IGF2BP2 expression and immunosuppressive cells, including M2 macrophages, Tregs, suggesting its role in shaping an immunosuppressive tumor microenvironment (TME). Building on these findings, we analyzed the IMvigor210 cohort to explore the relationship between IGF2BP2 expression and immunotherapy response. Patients with high IGF2BP2 expression exhibited poorer responses to ICIs compared to those with low expression, as shown by differences in response proportions. This highlights the potential utility of IGF2BP2 as a predictive biomarker for immunotherapy efficacy in BC. Notably, previous studies have implicated IGF2BP2 in modulating the TME through macrophage polarization and immune cell function in other cancers, such as colorectal cancer [55, 56]. These findings align with our results, further supporting the role of IGF2BP2 in immune regulation within the TME.

Next, we observed that IGF2BP2 is highly expressed in BC epithelial cells, particularly in GE‐9, which emerged as a distinct subpopulation with high CNV levels and immunosuppressive properties. To explore the interaction between GE‐9 and other cell types in the tumor microenvironment (TME), we integrated NicheNet and SCENIC analyses. NicheNet ligand‐receptor analysis identified SOX4 as a key regulator of ligand‐receptor interactions between GE‐9 and monocyte/macrophage populations, with IL6 and TGFB1 being among the top ligands predicted to influence monocyte/macrophage behavior [45]. Additionally, SCENIC transcription factor activity analysis highlighted BACH1 and XBP1 as critical transcriptional regulators in GE‐9, supporting their role in cytokine‐mediated modulation of the TME [46, 47]. These computational findings were further supported by co‐culture experiments. When IGF2BP2 expression was silenced in UM‐UC‐3 cells via siRNA, the recruitment of macrophages in co‐culture assays was significantly reduced compared to control cells, suggesting that IGF2BP2 plays a pivotal role in driving macrophage recruitment. RNA‐seq analysis of IGF2BP2‐knockdown cells revealed a marked downregulation of cytokines, including CCL2, which is known to regulate monocyte and macrophage polarization and infiltration [57, 58]. Recent studies have further demonstrated that IGF2BP2 can bind to and stabilize ZNF281 mRNA, subsequently enhancing CCL2 expression via ZNF281‐dependent transcriptional regulation [59].

Then, to gain insight into the types of macrophages recruited by IGF2BP2, we conducted immunofluorescence on BC samples. We observed that samples with high IGF2BP2 expression showed significant accumulation of CD14 and CD68 cells, markers of monocytes and macrophages, respectively. These results are consistent with the co‐culture experiments referring to macrophage recruitment. Meanwhile, the expression of CD163, a marker gene of M2 macrophage, is high in high‐expression IGF2BP2 group in TCGA‐BLCA and IMvigor210. Furthermore, both M2 score and angiogenesis score are significantly high in high‐IGF2BP2 group in TCGA‐BLCA and IMvigor210 cohort. These findings suggest that IGF2BP2‐expressing epithelial cells in BC may recruit M2‐like macrophages and promote angiogenesis. Previous studies have highlighted the complex heterogeneity of macrophage functions in the TME, extending beyond the classical M1/M2 paradigm [60]. Our findings add to this by demonstrating a positive correlation between IGF2BP2 expression and the infiltration of SPP1+ macrophages, a subtype known to promote immunosuppression and poor prognosis [61]. Additionally, high expression IGF2BP2 and SPP1+ macrophages are associated with poor overall survival in BC patients. Based on these results, we speculate that IGF2BP2‐expressing epithelial cells enhance the recruitment of SPP1+ macrophages, contributing to an immunosuppressive TME that impairs effective immunotherapy responses. Targeting IGF2BP2 in combination with immunotherapy could potentially counteract this immunosuppression and improve treatment outcomes.

There are several limitations to this study. First, our research primarily focuses on the transcriptomic role of IGF2BP2 in the BC microenvironment. While our findings suggest potential interactions between IGF2BP2 and monocyte–macrophage populations, further molecular studies are required to validate these interactions and elucidate the underlying mechanisms. Second, although differential cytokine expression was observed in IGF2BP2 knockdown experiments, further investigations are needed to clarify the precise molecular pathways through which IGF2BP2 regulates chemokine secretion and immune cell recruitment. Lastly, our study did not include direct profiling of m6A modifications but instead focused on the expression of m6A regulators as a surrogate for understanding their role in modulating the tumor microenvironment and clinical outcomes. Future studies incorporating genome‐wide m6A profiling could provide more comprehensive insights into the epitranscriptomic landscape of BC.

## 5 Conclusion

In conclusion, we demonstrate that IGF2BP2 regulates macrophage infiltration, contributing to changes in the composition of the TME in BC, leading to a suppressive and immunosuppressive microenvironment. These alterations may lead to varied responses to immunotherapy in BC. Given its role in shaping the TME and influencing immune cell infiltration, IGF2BP2 may serve as a potential therapeutic target for enhancing the efficacy of immunotherapy in bladder cancer. Targeting IGF2BP2 in combination with current immunotherapies could provide a novel strategy to overcome immune resistance and improve patient outcomes.

## Author Contributions

**Jianpeng Li:** conceptualization (equal), data curation (equal), formal analysis (equal), methodology (equal), software (equal), visualization (equal), writing – original draft (equal). **Yunzhong Jiang:** data curation (equal), formal analysis (equal), software (equal), validation (equal). **Minghai Ma:** data curation (equal), resources (equal), validation (equal). **Lu Wang:** validation (equal), writing – original draft (supporting), writing – review and editing (supporting). **Minxuan Jing:** investigation (supporting), methodology (supporting), writing – original draft (supporting), writing – review and editing (supporting). **Zezhong Yang:** validation (supporting), writing – original draft (supporting), writing – review and editing (supporting). **Lizhao Wang:** investigation (supporting), methodology (supporting), writing – review and editing (supporting). **Quanpeng Qiu:** investigation (supporting), methodology (supporting), writing – review and editing (supporting). **Rundong Song:** writing – original draft (supporting), writing – review and editing (supporting). **Yuanchun Pu:** writing – review and editing (equal). **Yuanquan Zhang:** writing – review and editing (supporting). **Nan Mei:** writing – original draft (supporting), writing – review and editing (supporting). **Mengzhao Zhang:** conceptualization (equal), project administration (equal), writing – review and editing (equal). **Jinhai Fan:** conceptualization (lead), funding acquisition (lead), project administration (lead), writing – review and editing (lead).

## Ethics Statement

All data used in this work are obtained from public databases and are publicly available. This work did not contain experiments on humans or animals; therefore, no ethical approval was obtained. The patients involved in the public database have received ethical approval. Our study about RNA‐seq and scRNA‐seq is based on open‐source data, so there are no ethical issues and other conflicts of interest.

## Conflicts of Interest

The authors declare no conflicts of interest.

## Supporting information


**Figure S1.** Single‐cell clustering and annotation of cell types in bladder cancer samples. (A) UMAP plot of single‐cell RNA sequencing data from 11 bladder cancer samples derived from the GSE190888 and GSE135337 datasets. Each color represents cells from a different sample, highlighting the diversity of cell populations across individual samples. (B) UMAP plot showing the clustering of single cells from bladder cancer samples into 11 distinct clusters (labeled 0–10) based on RNA expression profiles. (C) Expression patterns of marker genes used for cell type annotation. EPCAM is highly expressed in epithelial cells, PTPRC identifies immune cells, CD3D marks T/NK cells, LYZ indicates myeloid cells, COL1A1 highlights fibroblast cells, and PECAM1 is specific for endothelial cells. (D) Heatmap generated by inferCNV displaying inferred copy number variations (CNVs) across epithelial cell subpopulations (GE‐1 to GE‐9).

**Table S1.** The clinical information of TCGA‐BLCA and IMvigor210 analyzed in this study.
**Table S2.** The information of 26 m6A methylation regulators.
**Table S3.** The information of the siRNA sequence.
**Table S4.** The cluster‐specific marker genes derived from the TCGA‐BLCA cohort.
**Table S5.** Marker genes for different cell types.
**Table S6.** Signature Genes for M2, Angiogenesis, and Macro_SPP1.

## Data Availability

All high‐throughput sequencing and clinical data can be obtained from UCSC Xena (https://xenabrowser.net/datapages/). The data of GSE190888, GSE171351, GSE13507, GSE31684, GSE32548 and GSE146726 can be obtained from Gene Expression Omnibus (http://www.ncbi.nlm.nih.gov/geo/). The IMvigor210 data was obtained from the R package “IMvigor210CoreBiologies.”
