# Supplementary material for: IGF2BP2 Shapes the Tumor Microenvironment by Regulating Monocyte and Macrophage Recruitment in Bladder Cancer
Source: Cancer Med. 2024 Dec 23;13(24):e70506. doi: 10.1002/cam4.70506 (PMC11664238; doi:10.1002/cam4.70506)
Supplement: Supplementary file 1 — Figure S1. Single‐cell clustering and annotation of cell types in bladder cancer samples. (A) UMAP plot of single‐cell RNA sequencing data from 11 bladder cancer samples derived from the GSE190888 and GSE135337 datasets. Each color represents cells from a different sample, highlighting the diversity of cell populations across individual samples. (B) UMAP plot showing the clustering of single cells from bladder cancer samples into 11 distinct clusters (labeled 0–10) based on RNA expression profiles. (C) Expression patterns of marker genes used for cell type annotation. EPCAM is highly expressed in epithelial cells, PTPRC identifies immune cells, CD3D marks T/NK cells, LYZ indicates myeloid cells, COL1A1 highlights fibroblast cells, and PECAM1 is specific for endothelial cells. (D) Heatmap generated by inferCNV displaying inferred copy number variations (CNVs) across epithelial cell subpopulations (GE‐1 to GE‐9). [file CAM4-13-e70506-s002.pdf]

Figure S1

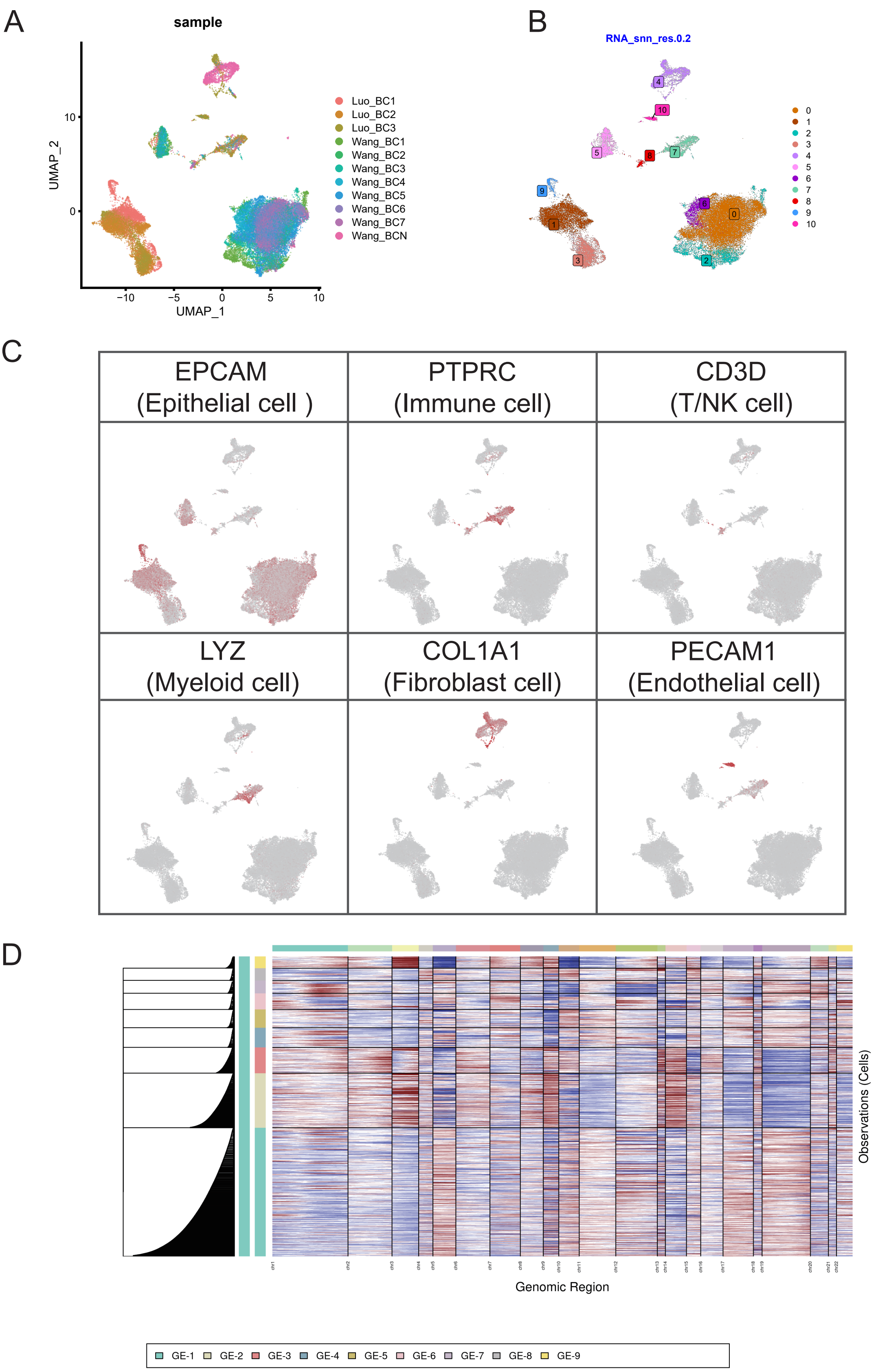

Figure S1. Single-cell clustering and annotation of cell types in bladder cancer samples.
(A) UMAP plot of single-cell RNA sequencing data from 11 bladder cancer samples derived from the GSE190888 and GSE135337 datasets. Each color represents cells from a different sample, highlighting the diversity of cell populations across individual samples. (B) UMAP plot showing the clustering of single cells from bladder cancer samples into 11 distinct clusters (labeled 0~10) based on RNA expression profiles. (C) Expression patterns of marker genes used for cell type annotation. EPCAM is highly expressed in epithelial cells, PTPRC identifies immune cells, CD3D marks T/NK cells, LYZ indicates myeloid cells, COL1A1 highlights fibroblast cells, and PECAM1 is specific for endothelial cells. (D) Heatmap generated by inferCNV displaying inferred copy number variations (CNVs) across epithelial cell subpopulations (GE-1 to GE-9).
